# Rapid Characterization of Virulence Determinants in *Helicobacter pylori* Isolated from Non-Atrophic Gastritis Patients by Next-Generation Sequencing

**DOI:** 10.3390/jcm8071030

**Published:** 2019-07-12

**Authors:** Frank Imkamp, Francis N. Lauener, Daniel Pohl, Philippe Lehours, Filipa F. Vale, Quentin Jehanne, Reinhard Zbinden, Peter M. Keller, Karoline Wagner

**Affiliations:** 1 Institute of Medical Microbiology, University of Zurich, 8006 Zurich, Switzerland; 2 Gastroenterology Division, University Hospital of Zurich, 8006 Zurich, Switzerland; 3 INSERM UMR1053, Bordeaux Research in Translational Oncology, BaRITOn, Université de Bordeaux, 33076 Bordeaux, France; 4 French National Reference Centre for Campylobacter and Helicobacter, Bordeaux Hospital, 33076 Bordeaux, France; 5 Host-Pathogen Interactions Unit, Research Institute for Medicine (iMed-ULisboa), Faculdade de Farmácia, Universidade de Lisboa, 1649-003 Lisboa, Portugal

**Keywords:** Helicobacter pylori, next generation sequencing, virulence, gastritis, *cagA*, *vacA*, *iceA*, *dupA*, *babA*, *babB*, *sabA*, *sabB*, *hopZ*, *hopQ*, *oipA*

## Abstract

*Helicobacter pylori* is a major human pathogen that causes a wide range of gastrointestinal pathology. Progression of *H. pylori* induced gastritis to more severe disease has been found to highly correlate with the array of virulence factors expressed by the pathogen. The objective of this study was twofold: first, to characterize the genetic diversity of *H. pylori* strains isolated from 41 non-atrophic gastritis patients in Switzerland, an issue that has not been investigated to date. And second, to assess the prevalence and sequence variation of *H. pylori* virulence factors (*cagA*, *vacA*, *iceA* and *dupA*) and genes encoding outer membrane proteins (OMPs; *babA*, *babB*, *sabA*, *sabB*, *hopZ*, *hopQ* and *oipA*) by whole genome sequencing (WGS) using an Illumina MiSeq platform. WGS identified high genetic diversity in the analyzed *H. pylori* strains. Most *H. pylori* isolates were assigned to hpEurope (95.0%, 39/41), and the remaining ones (5.0%, 2/41) to hpEastAsia, subpopulation hspEAsia. Analysis of virulence factors revealed that 43.9% of the strains were *cagA*-positive, and the *vacA* s1 allele was detected in 56.0% of the isolates. The presence of *cagA* was found to be significantly associated (*P* < 0.001) with the presence of *vacA* s1, *babA2* and *hopQ* allele 1 as well as expression of *oipA*. Moreover, we found an association between the grade of gastritis and *H. pylori* abundance in the gastric mucosa, respectively and the presence of *cagA*, *vacA* s1 and *hopQ* allele 1. Among our 41 gastritis patients, we identified seven patients infected with *H. pylori* strains that carried a specific combination of virulence factors (i.e., *cagA*, *vacA* s1 allele and *babA2* allele), recently implicated in the development of more severe gastrointestinal pathology, like peptic ulcer disease and even gastric cancer. To this end, WGS can be employed for rapid and detailed characterization of virulence determinants in *H. pylori*, providing valuable insights into the pathogenic capacity of the bacterium. This could ultimately lead to a higher level of personalized treatment and management of patients suffering from *H. pylori* associated infections.

## 1. Introduction

*Helicobacter pylori* is a major human pathogen that causes chronic inflammation of the gastric mucosa. While the majority of infected individuals remain asymptomatic, some develop severe gastrointestinal pathology like peptic ulcer disease (10–20%) or even gastric cancer (1–2%) [1,2].

Previous studies have shown that *H. pylori* strains display different degrees of virulence that determine their pathogenicity and potential progression of disease [3,4]. Understanding the factors that affect the progression of asymptomatic *H. pylori* infection to clinical disease is of utmost importance for efficient patient management.

The *H. pylori* genome encodes several potential virulence factors. One of the most important, the cytotoxin-associated gene (*cagA*) has been associated with mucosal inflammation and a more severe clinical outcome of *H. pylori*-associated infection [5]. The cytotoxin encoded by the vacuolating cytotoxin gene (*vacA*) damages epithelial cells by formation of vacuoles, initiates a proinflammatory response and specifically inhibits T-cell activation and proliferation [6,7]. VacA secretion by *H. pylori* has been reported to manipulate the autophagy pathway of its host, thereby facilitating intracellular survival and persistence of *H. pylori* in the gastric mucosa [8]. Although all *H. pylori* strains harbor *vacA*, sequence variations at the signal region (s1a, s1b and s2) and the middle region (m1 and m2) of the gene are associated with different levels of vacuolating activity of the encoded cytotoxin. Specifically, strains carrying *vacA* s1a/m1 exhibit more cytotoxic activity than strains with *vacA* s1b/m1, whereas *vacA* s2/m2 is not associated with any cytotoxic activity [3,9]. Another putative virulence factor present in virtually all *H. pylori* strains is encoded by *iceA*, which occurs in two allelic forms, namely *iceA1* and *iceA2* [10]. Expression of *iceA1* is upregulated upon contact of *H. pylori* with human gastric epithelial cells and has been associated with peptic ulcer disease [10,11]. In contrast, *iceA2* seems to not be involved in gastrointestinal pathology. Another marker indicative for the risk of gastric cancer development is the duodenal ulcer-promoting gene (*dupA*), which induces proinflammatory cytokine secretion by mononuclear cells [12,13,14]. *H. pylori* strains containing *dupA* have been associated with a reduced risk of developing gastric atrophy and gastric cancer compared to strains lacking this gene [15,16].

At the initiation of the infection process, *H. pylori* adheres to the gastric mucosa and mediates a gastric inflammatory response. More than 30 outer membrane proteins (OMPs) have been identified in *H. pylori*, and most of them are involved in bacterial adherence [17]. Production of OMPs is either regulated through recombination (gene conversion) or by slipped strand mispairing (phase variation) within CT dinucleotide repeat regions located at the 5’-end of the respective gene [17,18]. Important OMPs that potentially determine *H. pylori* pathogenicity are SabA, SabB, BabA, BabB, OipA, HopZ and HopQ. Expression of these OMPs may contribute to gastrointestinal disease by mediating *H. pylori* adherence, enhancing the activity of the *cag*-pathogenicity island, influencing *H. pylori*-induced signaling in host cells, or stimulating proinflammatory immune responses (e.g., [19,20]).

Virulence of *H. pylori* is commonly determined by targeted polymerase chain reaction (PCR) amplification of the genes mentioned above. However, this approach has serious drawbacks. First, multiple PCR reactions are needed to cover all known virulence genes and their variants, making a thorough characterization of *H. pylori* isolates cumbersome. Second, PCR amplification of the target region may be impaired, when mismatches occur in the primer binding sites. Moreover, high numbers of PCR amplification cycles may lead to artifacts in the amplified target sequence, potentially leading to wrong assumptions regarding the presence or structural variation of OMPs. In contrast, whole genome sequencing (WGS) enables relatively fast deep sequencing of bacterial genomes, and thus, is increasingly used in molecular diagnostics. Genomic information gained by WGS can be used to investigate bacterial antibiotic resistance (based on resistance genes and drug resistance mutations) and bacterial diversity and pathogenicity (based on the presence and expression of virulence factors identified in the bacterial genome). Only recently, we have described the clinical use of WGS for the prediction of drug resistance phenotypes in *H. pylori* [21].

In this follow-up study, we aimed to demonstrate that WGS data of *H. pylori* isolates also allows for a rapid characterization of virulence factors and the assessment of a strain’s pathogenic capacity. Since the frequency of virulence determinants in *H. pylori*, and their association with clinically relevant gastric disease appears to vary considerably between different geographic regions, we aimed to determine the frequency of virulence factors (*cagA*, *vacA*, *iceA* and *dupA*) and genes encoding OMPs (*babA*, *babB*, *sabA*, *sabB*, *hopQ*, *hopZ* and *oipA*) in Swiss patients suffering from non-atrophic gastritis.

## 2. Materials and Methods

### 2.1. Histology of Gastric Biopsy Specimens

Gastric antral and corpus specimens were taken with single-use forceps biopsy in accordance with the Sydney system [22]. Specifically, specimens were obtained from the lesser and greater curvature within maximum 3 cm of the pylorus, from the lesser curvature approximately 4 cm proximal to the angulus, from the middle lesser curvature (corpus) and incisura angularis. Formalin-fixed paraffin-embedded biopsies were cut with 2 mm thickness and routinely processed using staining with eosin/hematoxylin dye (Tissue-Tek Prisma™ 1, Sakura, Netherlands). Standard Giemsa staining was performed for assessment of *H. pylori* infection (Tissue-Tek Prisma™ 2). Quantitative and qualitative grading of gastritis was performed using the Sydney criteria: *H. pylori* positive/absent, presence of neutrophils and mononuclear cells, atrophy of antrum and/or corpus, presence of intestinal metaplasia [22]. Gastritis was graded as mild, moderate or marked, and the abundance of *H. pylori* was classified in the gastric biopsy specimens as few/some (+), abundant (++) and highly abundant (+++; Table S1).

### 2.2. Clinical H. pylori Isolates, Culture Methods and Phenotypic Drug Susceptibility Testing

*H. pylori* was isolated from multiple gastric antral and corpus biopsy specimens sent to the Institute of Medical Microbiology (IMM), University of Zurich, between January 2013 and December 2017 for culture-based phenotypic drug susceptibility testing (DST) after failed *H. pylori* eradication therapy. 

Clinical eradication was defined as a negative urea breath test or clinical impression of cure (symptom relief) on follow up assessments. Eradication therapy failure was defined as persistent positive urea breath test, repeat biopsy, or persistence of symptoms of dyspepsia in follow up assessments.

For this study, 41 *H. pylori* strains were incubated on in-house produced *Brucella* agar plates (with 5% horse blood) for 3 days at 37 °C under microaerobic conditions (90% N_2_, 5% CO_2_, 5% O_2_) using a gas generator (CampyGen, Thermo Scientific, Waltham, MA, USA). After 3 days, single *H. pylori* colonies were subcultured on *Brucella* agar plates and then stored at −80 °C in 3% skim milk (Difco Laboratories, Detroit, USA) supplemented with 5% glucose (Difco Laboratories). *H. pylori* isolates were stored at −80 °C in the bacterial strain collection of the IMM. In this study, *H. pylori* culture was done to perform culture-based phenotypic DST and DNA extraction for WGS.

*H. pylori* cultures were adjusted to a McFarland standard of 3 [23]. Phenotypic DST was performed on Mueller Hinton agar plates containing 5% horse blood (bioMérieux), using the following E-Tests^®^ (bioMérieux): amoxicillin (0.016–256 mg/L), clarithromycin (0.016–256 mg/L), metronidazole (0.016–256 mg/L), levofloxacin (0.016–32 mg/L), rifampicin (0.016–32 mg/L) and tetracycline (0.016–32 mg/L). Agar plates were incubated under microaerobic conditions at 37 °C for 3 days. Subsequently, MICs were determined by analysing the agar plates using a light microscope (Leica M80, Leica Microsystems, Heerbrugg, Switzerland). Susceptibility interpretation was performed according to the European Committee on Antimicrobial Susceptibility Testing (EUCAST) [24], except for rifampicin for which the susceptibility interpretation (MIC: S ≤ 4; R > 4) was done according to Hays et al. [25] and the Comité de l’Antibiogramme de la Société Française de Microbiologie (CASFM)/EUCAST recommendations for *H. pylori* [26].

### 2.3. DNA Extraction, Library Preparation and Sequencing of H. pylori Strains

DNA extraction from *H. pylori* cultures was performed with the DNeasy^®^ UltraClean^®^ Microbial kit (Qiagen, Hilden, Germany), following the manufacturer’s recommendations. Library preparation was done using the Qiagen^®^ QIAseq FX DNA kit (Qiagen, Hilden, Germany), according to the manufacturer’s recommendations with 6 min of fragmentation time. Sequencing library quality and size distribution (range of 300 bp to 700 bp; median of 500 bp) was analyzed on a fragment analyzer automated CE system (Advanced Analytical Technologies Inc., Heidelberg, Germany), according to the manufacturer’s instructions using the fragment analyzer 474 HS NGS kit. Sequencing libraries were pooled in equimolar concentrations and paired-end sequenced (2 × 150 bp) on an Illumina MiSeq platform (Illumina^®^, San Diego CA, USA). 

### 2.4. Bioinformatic and Statistical Analysis

Raw sequencing reads (fastq) were filtered and trimmed using the FASTQ trimmer tool of the FASTX-Toolkit (Hannon Lab, Cold Spring Harbour Laboratories) applying a threshold PHRED score of 25. In order to detect the presence and variants of different virulence genes, fastq files were analyzed using the ARIBA pipeline [27], querying a custom-made database of gene sequences derived from *H. pylori* strains available in the NCBI database (https://www.ncbi.nlm.nih.gov; Table S2). Contingency analysis was performed using the chi-square test (*X*^2^) of independence. A level of *p* < 0.05 was considered statistically significant. Statistics and data visualization was done in R [28].

### 2.5. Genotyping and Phylogenetic Analysis

**Figure 1:**
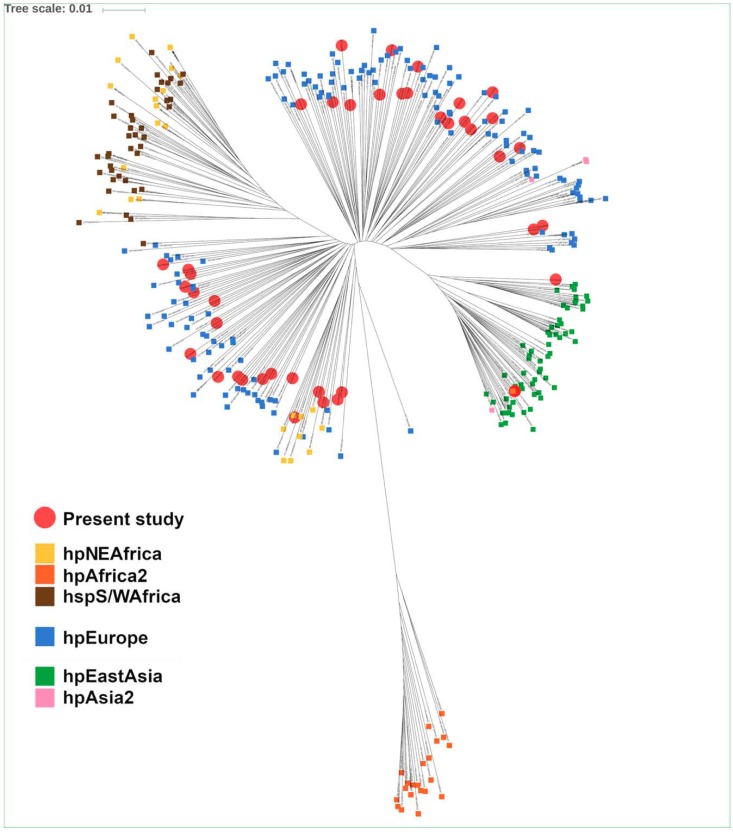
Phylogenetic analysis of the 41 *H. pylori* strains isolated from non-atrophic gastritis patients (Neighbor-joining tree-Kimura 2-parameter). Isolates representative of *H. pylori* populations are identified with colored squares and were retrieved from PubMLST. *H. pylori* isolates included in the present study are highlighted with red circles, and cluster mainly (95%) with hpEurope, but also with hpEastAsia (5%).

Phylogeography and population assignment was performed on the basis of seven housekeeping genes (*atpA*, *efp*, *trpC*, *ppa*, *mutY*, *yphC* and *ureI*). Each gene sequence was extracted from the WGS data of the 41 *H. pylori* strains using standard nucleotide BLAST. Additionally, 315 *H. pylori* strains with known origins available at PubMLST (http://pubmlst.org/helicobacter/), originally described by Falush et al. [29] were included in the analysis. A multi FASTA file was created, containing the concatenation of the seven housekeeping genes for each *H. pylori* strain. Sequences were aligned using the MUSCLE alignment algorithm. Newick tree format was generated using the neighbor -joining algorithm of the MEGA software [30], and a phylogenetic tree was constructed using the iTOL platform (https://itol.embl.de) (Figure 1). In addition, for each housekeeping gene, one multi FASTA file was created. Alleles of these 7 genes were aligned using MUSCLE. Alignment results were merged into one file and converted to the input format of STRUCTURE using xmfa2struct tool (Xavier Didelot, University of Warwick, Coventry, UK). Population assignment of the 41 *H. pylori* strains was performed with STRUCTURE [31,32], using the “no admixture model” and the 315 strains of PubMLST as training isolates (K = 20,000 iterations and a burn-in period of 20,000 iterations). Furthermore, the “admixture model” of STRUCTURE was performed to estimate the proportion of introgression from other populations in similar conditions. Finally, the subpopulation of hpEastAsia isolates were determined using the “no admixture model” of STRUCTURE and 121 strains from hpEastAsia populations (21 hspAmeridin, 50 hspEAsia and 50 hspMaori) from pubMLST as training isolates (K = 3 and 20,000 iterations and a burn-in period of 20,000 iterations).

### 2.6. Ethics Approval, Consent to Participate and Consent for Publication

The study was conducted according to good laboratory practice and in accordance with the Declaration of Helsinki and national and institutional standards. The Swiss act on medical research involving human subjects does not apply to this study as solely bacterial strains and anonymized patient-related data were used in this study. Thus, no consent for publication is required.

## 3. Results and Discussion

### 3.1. Patient Demographics and H. pylori Epidemiology

The patients’ demographics are summarized in Table 1. Secondary antibiotic resistance to clarithromycin and metronidazole, two antibiotics commonly used in empiric *H. pylori* eradication regimens, was high (85% and 73%; Table 1). The high drug resistance rate is to be expected given the “test-and-treat” strategy employed in Switzerland that only recommends endoscopy and culture-based phenotypic DST after a failed *H. pylori* eradication attempt [33]. Resistance to levofloxacin was 29% and to rifampicin 2%, whereas no resistance to tetracycline and amoxicillin was observed. 

Some *H. pylori* strains were resistant to multiple antibiotics (Table S1). Patient’s age did not correlate with increased antibiotic resistance in *H. pylori* (*P* > 0.05) as has been recently described for clarithromycin resistance in *Mycoplasma* sp. [34].

Most of the 41 *H. pylori* isolates were assigned to hpEurope, matching host ethnicity and the geographical area of *H. pylori* isolation. Additionally, hpEurope isolates showed scarce signs of introgression from hpAfrica1, which is consistent with Central European *H. pylori* isolates, supporting previous observations [35,36]. The remaining two isolates were assigned to hpEastAsia, subpopulation hspEAsia (Figure 1). Interestingly these strains were isolated from a Chinese and a Hispanic patient, respectively. While the former presented no signs of introgression from other populations, the latter displayed evidence of introgression from hpEurope, which agrees with the admixture pattern frequently found in Latin American isolates [35,36].

### 3.2. Presence of Virulence Determinants in H. pylori Isolates

**Figure 2:**
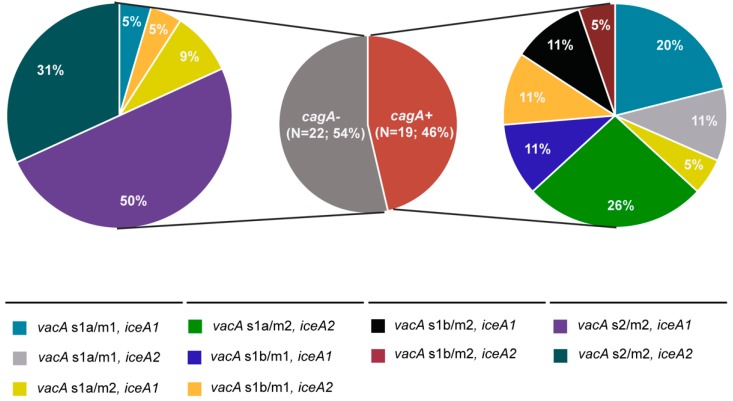
Determination of the presence of *cagA*, *vacA* and *iceA* in 41 *H. pylori* strains isolated from patients with non-atrophic gastritis.

A comprehensive characterization of the 41 *H. pylori* isolates was done based on WGS data. First, we focused on virulence-associated genes, namely *cagA*, *vacA*, *iceA* and *dupA*. Out of 41 *H. pylori* strains, 19 (46.3%) carried the *cagA* gene (Figure 2). The importance of determining the presence of *cagA* in clinical *H. pylori* isolates is underlined by previous studies that have consistently reported a higher risk for the development of premalignant lesions and even gastric cancer in patients infected with *cagA*-positive *H. pylori* strains (e.g., [37,38,39]). The number of *cagA*-positive *H. pylori* strains differs by geographic region, such as 60.0% in adult gastritis patients in Germany [39], 47.1% in the Netherlands [11], 66.0% in Finnish gastritis patients [40], 66.0%, 49.7% and 55.6% in France, Italy and Portugal, respectively [37,41,42], and 50.0% to 83.0% in Eastern European adult gastritis patients [38,43,44]. In the present study, the number of *H. pylori* strains carrying *cagA* (*N* = 19; 46.3%) closely relates to rates reported from these studies and agrees well with the ethnicity of the patient population, of which the majority has a Caucasian origin.

The majority of *H. pylori* isolates (23/41; 56%) harbored the *vacA* s1 allele. Specifically, the *vacA* s1a allele was detected in 15/41 (36.6%) *H. pylori* strains (7 s1a/m1, 8 s1a/m2) and the *vacA* s1b allele in 8/41 *H. pylori* strains (5 s1b/ml, 3 s1b/m2). The remaining 18 (43.9%) isolates harbored the *vacA* s2/m2 allele. The predominance of the *vacA* s1 allele found in this study is congruent with results from other European studies [11,37,38,39,44,45].

Recent studies have reported an association between the presence of *cagA* and the *vacA* s1 allele in *H. pylori* [6,9,46]. In accordance with these findings, all *cagA*-positive *H. pylori* strains harbored either the *vacA* s1a or s1b allele (6 s1a/m1, 6 s1a/m2, 4 s1b/m1 and 3 s1b/m2; Figure 2) in this study (X^2^ = 24.489, *P* < 0.001). Among the *vacA* s1 alleles, *vacA* s1/m1 has been found to be more toxic for epithelial cells than *vacA* s1/m2 [47], and has been therefore associated with benign ulcers disease and a higher risk for gastric cancer development [11,39,48,49,50]. In contrast, *vacA* s2/m2 strains are virtually nontoxic [3], and therefore, are rarely associated with gastrointestinal pathology [6,9,19]. In accordance, none of the 19 *cagA*-positive *H. pylori* strains harbored the *vacA* s2 allele (Figure 2).

In all 41 *H. pylori* strains, *iceA* was detected. The *iceA1* allele was identified in 23/41 (56.1%) and the *iceA2* allele in 18/41 (43.9%) *H. pylori* isolates (Figure 2). This finding is in accordance with other studies from European countries that reported on a predominance of *iceA1*-positive *H. pylori* strains in adult gastritis patients (e.g., [38,51,52]). The role of *iceA* and its contribution to gastrointestinal disease is somewhat controversial. Two reports suggested an association of *iceA1* with higher acute inflammation of the gastric mucosa and ulcers disease [10,11], while other studies could not confirm the association of *iceA1* with clinically relevant disease (e.g., [53,54]). This is in line with our observation that *iceA1* and *iceA2* were equally distributed (9 *iceA1 vs* 10 *iceA2*) among the 19 *cagA*-positive *H. pylori* isolates (*P* > 0.05; Figure 2).

The *dupA* gene was detected in 10/41 (24.4%) *H. pylori* isolates. Previous studies suggested its association with enhanced gastric inflammation, which could be due to *dupA*-mediated induction of proinflammatory cytokine secretion by mononuclear cells [12,15]. Thus, DupA appears to act similar to other proinflammatory virulence factors, including factors encoded by the *cag*-pathogenicity island and OipA [13,14,55]. Interestingly, *H. pylori* strains lacking *dupA* have been associated with an increased risk of developing duodenal ulcers or gastric cancer [15,16]. In this study, *dupA* was not more prevalent in *cagA*-positive than *cagA*-negative *H. pylori* strains (*P* > 0.05). In line with our results, Zhang et al. [56] found no association between the presence of *dupA* and *cagA*, *vacA*, *iceA* and *babA* alleles. This is in contrast to recent findings indicating a close association between *cagA* and *vacA* s1/m1, respectively, and *dupA* in patients with chronic gastritis or duodenal ulcers [57,58,59].

**Figure 3:**
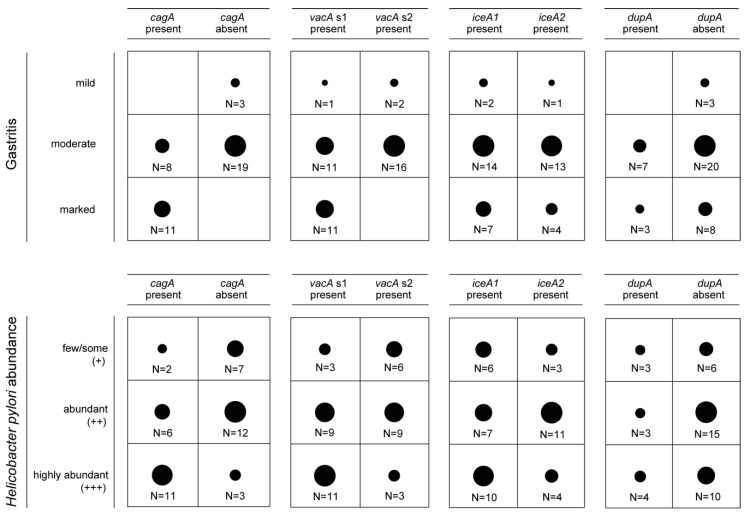
Assessment of the association between the presence of *cagA*, *vacA*, *iceA* and *dupA* and the severity of gastritis and *H. pylori* abundance in gastric biopsy specimens.

Moreover, we determined the association between the presence of *cagA*, *vacA*, *iceA* and *dupA* and the grade of gastritis and *H. pylori* abundance in the gastric mucosa, respectively. *CagA* was more abundant in *H. pylori* strains from gastritis patients with moderate or marked gastritis and patients that had a dense *H. pylori* colonization in the gastric mucosa (*P* < 0.01; Figure 3). For *vacA* allele s1, a significant association was only found with the severity of gastritis (*P* < 0.01), while *vacA* s1 was not significantly more prevalent in the gastric mucosa of patients with highly abundant *H. pylori* (*P* = 0.08). Nevertheless, a trend towards higher *vacA* s1 prevalence in *H. pylori* strains from gastric biopsies with dense *H. pylori* colonization could be observed. In contrast, neither the presence of *dupA* nor the presence of *iceA1* in *H. pylori* was associated with more severe gastritis or more dense *H. pylori* colonization in the gastric mucosa (*P* > 0.05; Figure 3).

Next, we investigated if more virulent *H. pylori* strains (i.e., strains carrying *cagA* and/or *vacA* s1) would be associated with clarithromycin resistance. Both virulence factors, *cagA* and *vacA* s1, were not associated with clarithromycin resistance (*P* > 0.05; Table S3).

### 3.3. Presence of Genes Encoding OMPs in H. pylori Isolates

OMP-mediated attachment to the gastric epithelium is crucial for the establishment of *H. pylori* associated infection and protects the pathogen from gastric acidity, as well as from displacement due to peristalsis. Sequence analysis of OMP encoding genes, specifically the CT-dinucleotide repeats at their 5’-ends, elicits their expression status (i.e., in-frame genes are expressed “status-on” and out-of-frame genes are not expressed “status-off”).

In-frame variants of *sabA* and *sabB* were found in 16/41 (39.0%) and 18/41 (43.9%) *H. pylori* isolates, respectively (“status-on”; Table 2). Expression of *sabA* has been found to be associated with an increased risk of chronic gastric inflammation, intestinal metaplasia, corpus atrophy and even gastric cancer [13,60,61]. A study by Mahadavi et al. [61] suggested that the presence of *sabA* is closely related to the presence of the *cag*-pathogenicity island and *babA*.

However, subsequent studies could not confirm this association [62,63,64]. Congruently, in the present study, in-frame *sabA* and *sabB* genes were not more prevalent in *cagA*-positive than in *cagA*-negative *H. pylori* strains (*P* > 0.05; Table 2).

The gene *hopZ* was expressed (in-frame gene; “status-on”) in 22/41 (53.7%) *H. pylori* strains. Accordingly, a study investigating HopZ expression in non-atrophic gastritis patients found *hopZ* “status-on” in 59% of patients [65]. HopZ is involved in adhesion to gastric epithelial cells [66,67], thereby providing *H. pylori* with a competitive fitness advantage for attachment to the gastric mucosa and initiation of infection [65,68]. However, HopZ was expressed in both, *cagA*-positive and negative *H. pylori* strains (*P* > 0.05; Table 2). Likewise, previous studies, found no correlation between HopZ expression (in-frame gene; “status-on”) and the presence of *cagA* [69]. Interestingly, a recent study demonstrated that patients infected with *H. pylori* strains harboring a combination of virulence factors, namely the *iceA1* allele, *sabA* “status-on” and *hopZ* “status-off”, had a 10 fold higher risk for developing a mucosa-associated lymphoid tissue (MALT) lymphoma [62], whereas hopZ status has not been found to correlate with the development of atrophic gastritis [65]. In this study, we identified 5 *H. pylori* strains with this specific combination of virulence factors (i.e., *iceA1* allele, *sabA* “status-on” and *hopZ* “status-off”; Table S1).

Analysis of the *oipA* sequence of the 41 *H. pylori* isolates identified in-frame variants in 20 strains (48.8%). OipA has been suggested to act as a disease-promoting factor, since expression of *oipA* correlates with increased production of interleukins (IL)-6, IL-8 and IL-11 [55,70]. In addition, OipA has been shown to mediate changes in motility, cytoskeletal reorganization and elongation of gastric epithelial cells by activation of the focal adhesion kinase [71]. These changes in host cells caused by the pathogen are ultimately important to establish infection. Thus, expression of *oipA* has been related to the development of gastrointestinal pathology including gastric cancer [13,55]. Clinical *H. pylori* isolates expressing OipA have been frequently found to additionally carry *cagA* and/or *vacA* s1 [13,55,72]. Concordantly, OipA expression (i.e., “status-on”) predominantly occurred in *cagA*-positive *H. pylori* strains (*X*^2^ = 7.03, *P* < 0.001; Table 2). Interestingly, *H. pylori* strains of East Asian origin have been found to exclusively harbor an in-frame *oipA* gene [55,72]. Congruently, the hspEAsia strain isolated from a gastritis patient with Chinese ethnicity expressed OipA, and additionally carried *cagA* and *vacA* s1 (Table 3).

The gene encoding the OMP HopQ was present in all *H. pylori* isolates. Among the 41 *H. pylori* isolates, *hopQ* allele 1 was found in ten strains (24.4%) that all were *cagA*-positive. *HopQ* allele 2 was identified in the remaining 31 *H. pylori* isolates (75.6%; 9 *cagA*-positive, 22 *cagA*-negative). These findings clearly indicate a positive association between *hopQ* allele 1 and the presence of *cagA* in *H. pylori* (*X*^2^ = 12.59, *P* < 0.001). This is congruent with the notion that *hopQ* allele 1 is required for maximal activity of the Cag-secretion system in *H. pylori* [73]. In addition, *hopQ* allele 1 has been associated with more invasive disease and associated with an increased risk for gastric cancer development [13].

Furthermore, we determined the association between the presence of *hopQ* alleles, the expression of *hopZ*, *oipA*, *sabA* and *sabB* and the grade of gastritis and *H. pylori* abundance in the gastric mucosa, respectively.

*HopQ* allele 1 was more abundant in *H. pylori* strains from gastritis patients with moderate or marked gastritis (*P* < 0.01), but was not significantly more prevalent in the gastric mucosa of patients with highly abundant *H. pylori* (*P* = 0.39; Tables S4 and S5). Expression of *oipA* was increased in patients with moderate or marked gastritis, but the difference was statistically not significant (*P* = 0.06). Expression of *hopZ*, *sabA* and *sabB* was neither associated with the grade of gastritis nor the abundance of *H. pylori* in the gastric mucosa (*P* > 0.05; Tables S4 and S5).

**Figure 4:**
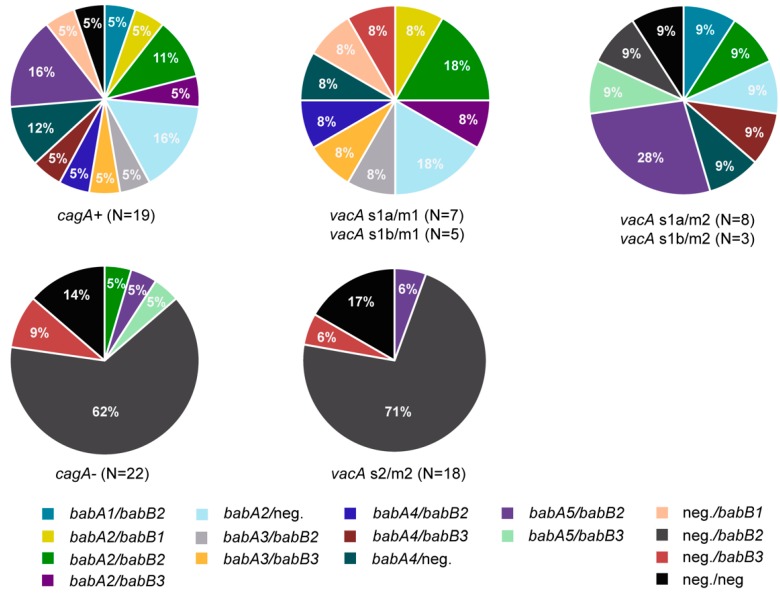
Determination of the presence of different *babA* and *babB* alleles and their association with *cagA* and *vacA* in 41 *H. pylori* strains isolated from patients with non-atrophic gastritis.

Finally, the 41 *H. pylori* isolates were analyzed with respect to the prevalence of the recently defined allele groups of *babA* (AD1 to AD5) and *babB* (BD1 to BD3) [20]. In total, 15/41 *H. pylori* isolates carried *babA*/*babB*, 5/41 only *babA*, 17/41 only *babB*, and 4/41 *H. pylori* strains neither carried *babA* nor *babB* (Figure 4). Seven *cagA*-positive *H. pylori* strains with *vacA* s1 alleles, additionally carried the *babA2* allele (Figure 4; Table 3). BabA2 has been previously associated with gastrointestinal pathology [60,74]. Moreover, other *babA* alleles were also found in *cagA*-positive *H. pylori* strains (*babA1*, *babA3*, *babA4*, *babA5*; *N* = 10; Figure 4). Interestingly, in only three out of 22 *cagA*-negative *H. pylori* strains, *babA* was found (1x *babA2* and 2x *babA5*; Figure 4). When we analyzed the association of *vacA* and *babA* alleles, we found different *babA* alleles in *H. pylori* strains with *vacA* s1, whereas, *babA5* was present in only one of 18 *H. pylori* strains with a *vacA* s2/m2 allele (Figure 4).

### 3.4. Presence of Multiple Virulence Determinants and OMPs in H. pylori Isolates

In this study, seven of 41 *H. pylori* strains carried multiple virulence factors, namely *cagA*, *vacA* s1 and *babA2*, whereas four of them additionally carried the *hopQ* allele 1 and expressed OipA, SabA and SabB (Table 3). The presence of specific virulence factors and expression of certain OMPs in *H. pylori* (i.e., *cagA*, *vacA* s1 and *babA2*) has been reported to have multiple effects on patients, such as (i) increased *H. pylori* density in the gastric mucosa [69], (ii) increased gastric inflammation and epithelial damage [49], and (iii) severity of *H. pylori* associated disease and development of peptic ulcers, gastric cancer and MALT lymphoma [49,60,62].

Moreover, geographic differences in the prevalence of virulence factors in *H. pylori* have been observed. In nearly all East Asian *H. pylori* isolates, strain-specific features linked to high gastric cancer risk (including the presence of *cagA*, *vacA* s1/m1 and *babA2*) have been detected [45,75,76]. In our patient population, only one patient with Chinese ethnicity was infected with a *H. pylori* isolate belonging to hspEAsia. The isolate carried *cagA*, *vacA* s1, *iceA1*, *babA2*, *hopQ* allele 1 and expressed OipA, SabA and SabB (Table 3). The predominance of strains harboring *cagA*, *vacA* s1/m1, and other strain-specific markers linked to gastric cancer may be one of the factors contributing to the high gastric cancer rate in East Asia. Congruently, *H. pylori* strains harboring few or none of these features have been reported to be less frequently associated with gastrointestinal pathology. *CagA*-negative *H. pylori* strains containing *vacA* s2 alleles and lacking *babA* are commonly found in the United States and Western Europe [3,45], and were found in the majority of our patient population.

Our study has several limitations: It was designed as a single center laboratory-based study using clinical *H. pylori* strains isolated from patients with non-atrophic gastritis. As Switzerland is a low prevalence country for gastric cancer, we could just include *H. pylori* strains from non-atrophic gastritis patients in this study. *H. pylori* strains exhibit a high degree of heterogeneity that supports the bacteria’s adaptation to and persistence in the gastric mucosa. Thus, some technical issues must be considered: First, multiple gastric biopsy specimens should be used for *H. pylori* culture in order to enhance sensitivity and detect *H. pylori* sub-populations. Second, multiple colonies should be picked from agar plates for DNA extraction and library preparation to not miss sub-populations. Third, *H. pylori* strains should be sequenced with sufficient coverage to detect all heterogeneity in the *H. pylori* population. Moreover, we had a rather small patient population (N=41). Consequently, we may have not been able to detect a statistically significant association between the presence of some virulence factors or their expression and the grade of gastritis as well as *H. pylori* abundance in the gastric mucosa. Thus, this association should be investigated in future studies in bigger patient cohorts.

## 4. Conclusion

In this study, we investigated the epidemiology of *H. pylori* and the presence of virulence genes in *H. pylori* strains isolated from 41 Swiss patients with non-atrophic gastritis. It has been previously reported that genetic determinants in the *H. pylori* genome and host factors play and important role in determining the clinical outcome of the *H. pylori* associated infection and the progression to more severe gastrointestinal pathology [1,2]. To evaluate if patients in our patient population may have an increased risk to develop more severe *H. pylori* associated disease, a WGS-based molecular analyses of *H. pylori* isolates in order to assess their virulence factor profiles was performed. *H. pylori* strains that carried *cagA*, *vacA* s1 and *hopQ* allele 1 were more abundant in patients with pronounced gastritis (grade moderate or marked) and led to more dense colonization in the gastric mucosa of these patients (i.e., *H. pylori* was abundant (++) or highly abundant (+++)). However, *cagA* and *vacA* s1 factors were not associated with drug resistance indicating that more virulent strains are not necessarily drug resistant. Moreover, we identified seven patients among our 41 patients that were infected with *H. pylori* strains that carried a combination of virulence factors reported to be associated with a higher risk for developing severe gastrointestinal pathology like duodenal ulcers and even gastric cancer. Current guidelines recommend to eradicate *H. pylori* in all individuals, even in patients with asymptomatic *H. pylori* gastritis [77]. To this end, next generation sequencing technology may be employed in clinical diagnostic laboratories for rapid monitoring of virulence determinants in *H. pylori* and assessment of an isolate’s pathogenic potential. This thorough characterization of *H. pylori* offers tremendous opportunities to improve individualized prognostic assessment, counselling, and follow-up care for patients. This could ultimately lead to a more personalized treatment and management of patients suffering from *H. pylori* associated infections.

## Figures and Tables

**Table 1:** Display of the epidemiological data of the patient population and drug resistance in *H. pylori* isolates.

**Age (years)**	Mean	45 ± 15
	Range	17–81
**Sex (Count)**	Female	25
	Male	16
**Ethnicity (Count)**	African	2
	Caucasian	36
	Chinese	1
	Hispanic	2
**Gastritis (Count)**	Mild	3
	Moderate	27
	Marked	11
***Helicobacter pylori* in gastric biopsy (Count)**	Few/Some (+)	9
	Abundant (++)	18
	Highly Abundant (+++)	14
**Drug-resistant *Helicobacter pylori* (Count)**	Amoxicillin	0
	Clarithromycin	35
	Metronidazole	30
	Levofloxacin	12
	Rifampicin	1
	Tetracycline	0

**Table 2:** Analysis of outer membrane protein encoding genes and their phase variations in 41 *H. pylori* strains isolated from patients with non-atrophic gastritis.

Gene	*cagA*-positive *H. pylori*		*cagA*-negative *H. pylori*		Difference between *cagA*-positive and *cagA*-negative *H. pylori* strains
	In-frame (“status-on”)	Out-of-frame (“status-off”)	In-frame (“status-on”)	Out-of-frame (“status-off”)	
*hopZ*	9	10	13	9	*P* > 0.05
*oipA*	14	5	6	16	*P* < 0.001
*sabA*	10	9	6	16	*P* > 0.05
*sabB*	10	9	8	14	*P* > 0.05

**Table 3:** The combination of virulence factors in seven presumably highly virulent *H. pylori* strains.

Patient	Age	Sex	Ethnicity	*cagA*	*vacA*	*iceA*	*babA*	*babB*	*dupA*	*hopZ*	*oipA*	*sabA*	*sabB*	*hopQ*
1	31	F	Caucasian	+	s1a/m1	iceA1	*babA2*	*babB2*	-	off	on	on	off	allele 1
2	50	M	Caucasian	+	s1a/m2	iceA2	*babA2*	*babB2*	-	on	on	off	off	allele 1
3	28	M	Chinese	+	s1a/m2	iceA1	*babA2*	-	-	off	on	on	on	allele 1
4	26	M	Caucasian	+	s1a/m1	iceA1	*babA2*	*babB3*	-	off	off	on	on	allele 2
5	54	M	Caucasian	+	s1a/m1	iceA1	*babA2*	-	-	on	on	on	on	allele 1
6	54	M	Caucasian	+	s1b/m1	iceA2	*babA2*	*babB1*		off	on	off	on	allele 2
7	50	F	Caucasian	+	s1b/m1	iceA2	*babA2*	-	+	off	off	off	off	allele 2

“on” indicates in-frame genes that are expressed; “off” indicates out-off-frame genes that are not expressed.

## Data Availability

Whole genome sequences of the *H. pylori* strains analyzed in this study are available on NCBI under accession numbers: RJHB00000000, RJFC00000000, RJFY00000000, RJGH00000000, RJGK00000000, RJGL00000000, RJGO00000000, RJGV00000000, RJGY00000000, RJHA00000000, RJHC00000000, RJHD00000000, RJHF00000000, RJHG00000000, RJHL00000000, RJHM00000000, RJHR00000000, RJHT00000000, RJHU00000000, RJHW00000000, RJHX00000000, RJIA00000000, RJIC00000000, RJID00000000, RJIE00000000, RJIH00000000, RJII00000000, RJIJ00000000, RJIK00000000, RJIL00000000, RJIO00000000, RJIP00000000, RJIQ00000000, RJIR00000000, RJIS00000000, RJIU00000000, RJIV00000000, RJIW00000000, RJIY00000000.

## Supplementary Materials

The following are available online at https://www.mdpi.com/2077-0383/8/7/1030/s1, Table S1: Summary of demographic patient information, endoscopic data and overview of virulence factors and phenotypic antibiotic resistance information of the *H. plori* isolates; virulence factors: a grey background indicates the presence of a virulence gene or that the virulence gene is expressed (“status-on”); antibiotic resistance information: a red background indicates resistance of *H. pylori* to the respective antibiotic. MIC, minimum inhibitory concentration, Table S2: Gene sequences derived from *H. pylori* strains available in the NCBI database used to detect the presence and variants of different virulence genes in the 41 *H. pylori* strains isolated from non-atrophic gastritis patients, Table S3: Association between the presence of *cagA* and *vacA* alleles and clarithromycin resistance, Table S4: Association between the abundance of *Helicobacter pylori* in the gastric mucosa and the expression of outer membrane proteins, Table S5: Association between the grade of gastritis and the expression of outer membrane proteins in *Helicobacter pylori*.

## References

[1] Wroblewski L.E., Peek R.M., Wilson K.T. (2010). *Helicobacter pylori* and gastric cancer: Factors that modulate disease risk. Clin. Microbiol. Rev..

[2] Jonaitis L., Pellicano R., Kupcinskas L. (2018). *Helicobacter pylori* and nonmalignant upper gastrointestinal diseases. Helicobacter.

[3] Atherton J.C., Cao P., Peek R.M., Tummuru M.K., Blaser M.J., Cover T.L. (1995). Mosaicism in vacuolating cytotoxin alleles of *Helicobacter pylori* association of specific vacA types with cytotoxin production and peptic ulceration. J. Biol. Chem..

[4] Blaser M.J., Perez-Perez G.I., Kleanthous H., Cover T.L., Peek R.M., Chyou P., Stemmermann G.N., Nomura A. (1995). Infection with *Helicobacter pylori* strains possessing *cagA* is associated with an increased risk of developing adenocarcinoma of the stomach. Cancer Res..

[5] Censini S., Lange C., Xiang Z., Crabtree J.E., Ghiara P., Borodovsky M., Rappuoli R., Covacci A. (1996). *Cag*, a pathogenicity island of *Helicobacter pylori*, encodes type I-specific and disease-associated virulence factors. Proc. Natl. Acad. Sci. USA.

[6] Atherton J.C. (2006). The pathogenesis of *Helicobacter pylori* induced gastro-duodenal diseases. Annu. Rev. Pathol..

[7] Camilo V., Sugiyama T., Touati E. (2017). Pathogenesis of *Helicobacter pylori* infection. Helicobacter.

[8] Greenfield L.K., Jones N.L. (2013). Modulation of autophagy by *Helicobacter pylori* and its role in gastric carcinogenesis. Trends Microbiol..

[9] Atherton J., Peek R., Tham K., Cover T., Blaser M. (1997). Clinical and pathological importance of heterogeneity in *vacA*, the vacuolating cytotoxin gene of *Helicobacter pylori*. Gastroenterology.

[10] Peek J.R., Thompson S.A., Donahue J.P., Tham K.T., Atherton J.C., Blaser M.J., Miller G.G. (1998). Adherence to gastric epithelial cells induces expression of a *Helicobacter pylori* gene, *iceA*, that is associated with clinical outcome. Proc. Assoc. Am. Phys..

[11] van Doorn L.J., Figueiredo C., Sanna R., Plaisier A., Schneeberger P., de Boer W., Quint W. (1998). Clinical relevance of the *cagA*, *vacA*, and *iceA* status of *Helicobacter pylori*. Gastroenterology.

[12] (2010). Hussein NR, Argent RH, Marx CK, Patel SR, Robinson K, Atherton JC: Helicobacter pylori dupA is polymorphic, and its active form induces proinflammatory cytokine secretion by mononuclear cells. J. Infect. Dis..

[13] Yamaoka Y., Ojo O., Fujimoto S., Odenbreit S., Haas R., Gutierrez O., El-Zimaity H.M., Reddy R., Arnqvist A., Graham D.Y. (2006). *Helicobacter pylori* outer membrane proteins and gastroduodenal disease. Gut.

[14] Ando T., Peek R.M., Lee Y.C., Krishna U., Kusugami K., Blaser M.J. (2002). Host cell responses to genotypically similar *Helicobacter pylori* isolates from United States and Japan. Clin. Diagn. Lab. Immunol..

[15] Lu H., Hsu P.I., Graham D.Y., Yamaoka Y. (2005). Duodenal ulcer promoting gene of *Helicobacter pylori*. Gastroenterology.

[16] Abadi A.T.B., Taghvaei T., Wolfram L., Kusters J.G. (2012). Infection with *Helicobacter pylori* strains lacking *dupA* is associated with an increased risk of gastric ulcer and gastric cancer development. J. Med. Microbiol..

[17] Tomb J.F., White O., Kerlavage A.R., Clayton R.A., Sutton G.G., Fleischmann R.D., Ketchum K.A., Klenk H.P., Gill S., Dougherty B.A. (1997). The complete genome sequence of the gastric pathogen *Helicobacter pylori*. Nature.

[18] Alm R.A., Ling L.S.L., Moir D.T., King B.L., Brown E.D., Doig P.C., Smith D.R., Noonan B., Guild B.C., Carmel G. (1999). Genomic-sequence comparison of two unrelated isolates of the human gastric pathogen *Helicobacter pylori*. Nature.

[19] Cover T.L. (2016). *Helicobacter pylori* diversity and gastric cancer risk. MBio.

[20] Pride D.T., Meinersmann R.J., Blaser M.J. (2001). Allelic variation within *Helicobacter pylori babA* and *babB*. Infect. Immun..

[21] Lauener F., Imkamp F., Lehours P., Buissonnière A., Benejat L., Zbinden R., Keller P., Wagner K. (2019). Genetic determinants and prediction of antibiotic resistance phenotypes in *Helicobacter pylori*. J. Clin Med..

[22] Dixon M.F., Genta R.M., Yardley J.H., Correa P. (1996). Classification and grading of gastritis: The updated Sydney system. Am. J. Surg. Pathol..

[23] McFarland J. (1907). The nephelometer: An instrument for estimating the number of bacteria in suspensions used for calculating the opsonic index and for vaccines. JAMA.

[24] EUCAST (2019). Breakpoint Tables for Interpretation of MICs and Zone Diameters, Version 9.0. http://www.eucast.org.

[25] Hays C., Burucoa C., Lehours P., Tran C.T., Leleu A., Raymond J. (2018). Molecular characterization of *Helicobacter pylori* resistance to rifamycins. Helicobacter.

[26] CASFM/EUCAST (2019). Breakpoint Tables for Interpretation of MICs and Zone Diameters, Version 1.0. www.sfm-microbiologie.org.

[27] Hunt M., Mather A.E., Sánchez-Busó L., Page A.J., Parkhill J., Keane J.A., Harris S.R. (2017). ARIBA: Rapid antimicrobial resistance genotyping directly from sequencing reads. Microb. Genom..

[28] R Development Core Team (2018). R: A Language and Environment for Statistical Computing.

[29] Falush D., Wirth T., Linz B., Pritchard J.K., Stephens M., Kidd M., Blaser M.J., Graham D.Y., Vacher S., Perez-Perez G.I. (2003). Traces of human migrations in *Helicobacter pylori* populations. Science.

[30] Tamura K., Stecher G., Peterson D., Filipski A., Kumar S. (2013). MEGA6: Molecular evolutionary genetics analysis version 6.0. Mol. Biol. Evol..

[31] Pritchard J.K., Stephens M., Donnelly P. (2000). Inference of population structure using multilocus genotype data. Genetics.

[32] Falush D., Stephens M., Pritchard J.K. (2003). Inference of population structure using multilocus genotype data: Linked loci and correlated allele frequencies. Genetics.

[33] Malfertheiner P., Megraud F., O’morain C., Gisbert J., Kuipers E., Axon A., Bazzoli F., Gasbarrini A., Atherton J., Graham D. (2017). Management of *Helicobacter pylori* infection—the Maastricht V/Florence consensus report. Gut.

[34] Wagner K., Imkamp F., Pires V., Keller P. (2019). Evaluation of Lightmix Mycoplasma macrolide assay for detection of macrolide-resistant *Mycoplasma pneumoniae* in pneumonia patients. Clin. Microbiol. Infect..

[35] Oleastro M., Rocha R., Vale F.F. (2017). Population genetic structure of *Helicobacter pylori* strains from Portuguese-speaking countries. Helicobacter.

[36] Thorell K., Yahara K., Berthenet E., Lawson D.J., Mikhail J., Kato I., Mendez A., Rizzato C., Bravo M.M., Suzuki R. (2017). Rapid evolution of distinct *Helicobacter pylori* subpopulations in the Americas. PLoS Genet..

[37] Figueiredo C., Van Doorn L.J., Nogueira C., Soares J., Pinho C., Figueira P., Quint W., Carneiro F. (2001). *Helicobacter pylori* genotypes are associated with clinical outcome in Portuguese patients and show a high prevalence of infections with multiple strains. Scand. J. Gastroenterol..

[38] Erzin Y., Koksal V., Altun S., Dobrucali A., Aslan M., Erdamar S., Dirican A., Kocazeybek B. (2006). Prevalence of *Helicobacter pylori vacA*, *cagA*, *cagE*, *iceA*, *babA2* genotypes and correlation with clinical outcome in Turkish patients with dyspepsia. Helicobacter.

[39] Miehlke S., Kirsch C., Agha-Amiri K., Günther T., Lehn N., Malfertheiner P., Stolte M., Ehninger G., Bayerdörffer E. (2000). The *Helicobacter pylori vacA* s1, m1 genotype and *cagA* is associated with gastric carcinoma in Germany. Int. J. Cancer.

[40] Heikkinen M., Mayo K., Megraud F., Vornanen M., Marin S., Pikkarainen P., Julkunen R. (1998). Association of CagA-positive and CagA-negative *Helicobacter pylori* strains with patients’ symptoms and gastritis in primary care patients with functional upper abdominal complaints. Scand. J. Gastroenterol..

[41] Audibert C., Janvier B., Grignon B., Salaüna L., Burucoa C., Lecron J.C., Fauchère J.L. (2000). Correlation between IL-8 induction, *cagA* status and *vacA* genotypes in 153 French *Helicobacter pylori* isolates. Res. Microbiol..

[42] Chiarini A., Calà C., Bonura C., Gullo A., Giuliana G., Peralta S., D’Arpa F., Giammanco A. (2009). Prevalence of virulence-associated genotypes of *Helicobacter pylori* and correlation with severity of gastric pathology in patients from western Sicily, Italy. Eur. J. Clin. Microbiol. Infect. Dis..

[43] Boyanova L., Markovska R., Yordanov D., Marina M., Ivanova K., Panayotov S., Gergova G., Mitov I. (2009). High prevalence of virulent *Helicobacter pylori* strains in symptomatic Bulgarian patients. Diagn. Microbiol. Infect. Dis..

[44] Andreson H., Loivukene K., Sillakivi T., Maaroos H.I., Ustav M., Peetsalu A., Mikelsaar M. (2002). Association of *cagA* and *vacA* genotypes of *Helicobacter pylori* with gastric diseases in Estonia. J. Clin. Microbiol..

[45] Van Doorn L.J., Figueiredo C., Mégraud F., Pena S., Midolo P., Queiroz D.M.D.M., Carneiro F., Vanderborght B., Maria Da Glória F.P., Sanna R. (1999). Geographic distribution of *vacA* allelic types of *Helicobacter pylori*. Gastroenterology.

[46] Yamaoka Y., Kodama T., Kita M., Imanishi J., Kashima K., Graham D.Y. (1998). Relationship of *vacA* genotypes of *Helicobacter pylori* to *cagA* status, cytotoxin production, and clinical outcome. Helicobacter.

[47] Letley D.P., Rhead J.L., Twells R.J., Dove B., Atherton J.C. (2003). Determinants of non-toxicity in the gastric pathogen *Helicobacter pylori*. J. Biol. Chem..

[48] Van Doorn L., Figueiredo C., Rossau R., Jannes G., Van Asbroeck M., Sousa J., Carneiro F., Quint W. (1998). Typing of *Helicobacter pylori vacA* gene and detection of *cagA* gene by PCR and reverse hybridization. J. Clin. Microbiol..

[49] Figueiredo C., Machado J.C., Pharoah P., Seruca R., Sousa S., Carvalho R., Capelinha A.F., Quint W., Caldas C., van Doorn L.J. (2002). *Helicobacter pylori* and interleukin 1 genotyping: An opportunity to identify high-risk individuals for gastric carcinoma. J. Natl. Cancer. Inst..

[50] Kidd M., Lastovica A., Atherton J., Louw J. (1999). Heterogeneity in the *Helicobacter pylori vacA* and *cagA* genes: Association with gastroduodenal disease in South Africa?. Gut.

[51] Miehlke S., Yu J., Schuppler M., Frings C., Kirsch C., Negraszus N., Morgner A., Stolte M., Ehninger G., Bayerdörffer E. (2001). *Helicobacter pylori vacA*, *iceA*, and *cagA* status and pattern of gastritis in patients with malignant and benign gastroduodenal disease. Am. J. Gastroenterol..

[52] Boyanova L., Yordanov D., Gergova G., Markovska R., Mitov I. (2010). Association of *iceA* and *babA* genotypes in *Helicobacter pylori* strains with patient and strain characteristics. Antonie Van Leeuwenhoek.

[53] Yamaoka Y., Kodama T., Gutierrez O., Kim J.G., Kashima K., Graham D.Y. (1999). Relationship between *Helicobacter pylori iceA*, *cagA*, and *vacA* status and clinical outcome: Studies in four different countries. J. Clin. Microbiol..

[54] Nishiya D., Shimoyama T., Fukuda S., Yoshimura T., Tanaka M., Munakata A. (2000). Evaluation of the clinical relevance of the *iceA1* gene in patients with *Helicobacter pylori* infection in Japan. Scand. J. Gastroenterol..

[55] Yamaoka Y., Kikuchi S., El–Zimaity H.M., Gutierrez O., Osato M.S., Graham D.Y. (2002). Importance of *Helicobacter pylori oipA* in clinical presentation, gastric inflammation, and mucosal interleukin 8 production. Gastroenterology.

[56] Zhang Z., Zheng Q., Chen X., Xiao S., Liu W., Lu H. (2008). The *Helicobacter pylori* duodenal ulcer promoting gene, *dupA* in China. BMC Gastroenterol..

[57] Pereira W.N., Ferraz M.A., Zabaglia L.M., de Labio R.W., Orcini W.A., Ximenez J.P.B., Neto A.C., Payão S.L.M., Rasmussen L.T. (2014). Association among *H. pylori* virulence markers *dupA, cagA* and *vacA* in Brazilian patients. J. Venom Anim. Toxins Incl. Trop. Dis..

[58] Arachchi H.J., Kalra V., Lal B., Bhatia V., Baba C., Chakravarthy S., Rohatgi S., Sarma P.M., Mishra V., Das B. (2007). Prevalence of duodenal ulcer-promoting gene (*dupA*) of *Helicobacter pylori* in patients with duodenal ulcer in North Indian population. Helicobacter.

[59] Gomes L.I., Rocha G.A., Rocha A.M., Soares T.F., Oliveira C.A., Bittencourt P.F., Queiroz D.M. (2008). Lack of association between *Helicobacter pylori* infection with *dupA*-positive strains and gastroduodenal diseases in Brazilian patients. Int. J. Med. Microbiol..

[60] Gerhard M., Lehn N., Neumayer N., Borén T., Rad R., Schepp W., Miehlke S., Classen M., Prinz C. (1999). Clinical relevance of the *Helicobacter pylori* gene for blood-group antigen-binding adhesin. Proc. Natl. Acad. Sci. USA.

[61] Mahdavi J., Sondén B., Hurtig M., Olfat F.O., Forsberg L., Roche N., Ångström J., Larsson T., Teneberg S., Karlsson K.A. (2002). *Helicobacter pylori* SabA adhesin in persistent infection and chronic inflammation. Science.

[62] Lehours P., Ménard A., Dupouy S., Bergey B., Richy F., Zerbib F., Ruskoné-Fourmestraux A., Delchier J.C., Mégraud F. (2004). Evaluation of the association of nine *Helicobacter pylori* virulence factors with strains involved in low-grade gastric mucosa-associated lymphoid tissue lymphoma. Infect. Immun..

[63] De Jonge R., Pot R.G., Loffeld R.J., Van Vliet A.H., Kuipers E.J., Kusters J.G. (2004). The functional status of the *Helicobacter pylori sabB* adhesin gene as a putative marker for disease outcome. Helicobacter.

[64] Sheu B.S., Odenbreit S., Hung K.H., Liu C.P., Sheu S.M., Yang H.B., Wu J.J. (2006). Interaction between host gastric Sialyl-Lewis X and *H. pylori* SabA enhances *H. pylori* density in patients lacking gastric Lewis B antigen. Am. J. Gastroenterol..

[65] Kennemann L., Brenneke B., Andres S., Engstrand L., Meyer T.F., Aebischer T., Josenhans C., Suerbaum S. (2012). In vivo sequence variation in HopZ, a phase-variable outer membrane protein of *Helicobacter pylori*. Infect. Immun..

[66] Peck B., Ortkamp M., Diehl K.D., Hundt E., Knapp B. (1999). Conservation, localization and expression of HopZ, a protein involved in adhesion of *Helicobacter pylori*. Nucleic Acid. Res..

[67] Yamaoka Y., Kudo T., Lu H., Casola A., Brasier A.R., Graham D.Y. (2004). Role of interferon-stimulated responsive element-like element in interleukin-8 promoter in *Helicobacter pylori* infection. Gastroenterology.

[68] Giannakis M., Backhed H.K., Chen S.L., Faith J.J., Wu M., Guruge J.L., Engstrand L., Gordon J.I. (2009). The response of gastric epithelial progenitors to *Helicobacter pylori* isolates obtained from Swedish patients with chronic atrophic gastritis. J. Biol. Chem..

[69] Yamaoka Y., Kita M., Kodama T., Imamura S., Ohno T., Sawai N., Ishimaru A., Imanishi J., Graham D.Y. (2002). *Helicobacter pylori* infection in mice: Role of outer membrane proteins in colonization and inflammation. Gastroenterology.

[70] Yamaoka Y., Kwon D.H., Graham D.Y. (2000). A Mr 34,000 proinflammatory outer membrane protein (*oipA*) of *Helicobacter pylori*. Proc. Natl. Acad. Sci. USA.

[71] Tabassam F.H., Graham D.Y., Yamaoka Y. (2008). OipA plays a role in *Helicobacter pylori*-induced focal adhesion kinase activation and cytoskeletal re-organization. Cell. Microbiol..

[72] Ando T., Peek R., Pride D., Levine S., Takata T., Lee Y.C., Kusugami K., Van der Ende A., Kuipers E., Kusters J. (2002). Polymorphisms of *Helicobacter pylori* HP0638 reflect geographic origin and correlate with *cagA* status. J. Clin. Microbiol..

[73] Belogolova E., Bauer B., Pompaiah M., Asakura H., Brinkman V., Ertl C., Bartfeld S., Nechitaylo T.Y., Haas R., Machuy N. (2013). *Helicobacter pylori* outer membrane protein HopQ identified as a novel T4SS-associated virulence factor. Cell. Microbiol..

[74] Yu J., Leung W., Go M., Chan M., To K., Ng E., Chan F., Ling T., Chung S., Sung J. (2002). Relationship between *Helicobacter pylori babA2* status with gastric epithelial cell turnover and premalignant gastric lesions. Gut.

[75] Ito Y., Azuma T., Ito S., Miyaji H., Hirai M., Yamazaki Y., Sato F., Kato T., Kohli Y., Kuriyama M. (1997). Analysis and typing of the *vacA* gene from *cagA*-positive strains of *Helicobacter pylori* isolated in Japan. J. Clin. Microbiol..

[76] Lai C.H., Kuo C.H., Chen Y.C., Chao F.Y., Poon S.K., Chang C.S., Wang W.C. (2002). High prevalence of *cagA*-and *babA2*-positive *Helicobacter pylori* clinical isolates in Taiwan. J. Clin. Microbiol..

[77] Fischbach W., Malfertheiner P., Jansen P.L., Bolten W., Bornschein J., Buderus S., Glocker E., Hoffmann J., Koletzko S., Labenz J. (2016). S2k-Leitlinie *Helicobacter pylori* und gastroduodenale Ulkuskrankheit. Zeitschrift für Gastroenterologie.

