# Supplementary material for: Rapid Characterization of Virulence Determinants in Helicobacter pylori Isolated from Non-Atrophic Gastritis Patients by Next-Generation Sequencing
Source: J Clin Med. 2019 Jul 12;8(7):1030. doi: 10.3390/jcm8071030 (PMC6678415; doi:10.3390/jcm8071030)
Supplement: Supplementary file 1 [file jcm-08-01030-s001.zip › jcm-548599-supplementary.docx]

**Supplementary Material**

Rapid characterization of virulence determinants in *Helicobacter pylori* isolated from non-atrophic gastritis patients by next generation sequencing

**Table 1.** Summary of demographic patient information, endoscopic data and overview of virulence factors and phenotypic antibiotic resistance information of the *H. pylori* isolates; virulence factors: a grey background indicates the presence of a virulence gene or that the virulence gene is expressed (“status-on”); antibiotic resistance information: a red background indicates resistance of *H. pylori* to the respective antibiotic. MIC, minimum inhibitory concentration.

**Table 2.** Gene sequences derived from *H. pylori* strains available in the NCBI database used to detect the presence and variants of different virulence genes in the 41 *H. pylori* strains isolated from non-atrophic gastritis patients.

| **Gene** | **Variant** | **NCBI accession number** |
| --- | --- | --- |
| *babA* |  | NC_000915.1:1317838-1320039 |
|  | AD1 | AF277904.1 |
|  | AD2 | AF277905.1 |
|  | AD3 | AF277906.1 |
|  | AD4 | AF277907.1 |
|  | AD5 | AF277938.1 |
| *babB* |  | NC_000915.1:947580-949706 |
|  | BD1 | AF277943.1 |
|  | BD2 | AF277959.1 |
| *cagA* |  | NC_000915.1:579921-583481 |
| *dupA* |  | NC_000915.1:459333-461756 |
| *hopZ* |  | NC_000915.1:5241-7145_ |
| *hopQ* | allele 1 | NC_000915.1:1243583-1245508 |
|  | allele 2 | NC_000915.1:1245964-1246665 |
| *iceA* |  | NC_000915.1:1286191-1286877 |
|  | iceA1 | AF459446.1 |
|  | iceA2 | JQ808069.1 |
| *oipA* |  | NC_000915.1:684774-685691 |
| *sabA* |  | NC_000915.1:779008-780897 |
| *sabB* |  | CP001173.1:741597-743513 |
| *vacA* |  | NC_000915.1:938415-942287 |
|  | m1 | U05676.1 |
|  | m2 | U05677.1 |
|  | s1 | LC187397.1 |
|  | s1a | AY840127.1 |
|  | s1b | DQ061143.1 |
|  | s2 | U29401.1 |

**Table 3.** Association between the presence of *cagA* and *vacA* alleles and clarithromycin resistance.

| **Virulence factor** | **Clarithromycin** | |
| --- | --- | --- |
|  | **resistant** | **susceptible** |
| *cagA* positive | 14 | 5 |
| *cagA* negative | 21 | 1 |
|  | | |
| *vacA* s1 | 18 | 5 |
| *vacA* s2 | 17 | 1 |

**Table 4.** Association between the abundance of *Helicobacter pylori* in the gastric mucosa and the expression of outer membrane proteins.

| **Outer membrane protein** | | *Helicobacter pylori* abundance in the gastric mucosa | | |
| --- | --- | --- | --- | --- |
|  |  | Few/Some (+) | Abundant (++) | Highly abundant (+++) |
| ***hopQ*** | allele 1 | 1 | 4 | 5 |
|  | allele 2 | 8 | 14 | 9 |
|  | | | | |
| *hopZ* | Status-on | 4 | 9 | 5 |
|  | Status-off | 5 | 9 | 9 |
|  | | | | |
| *oipA* | Status-on | 6 | 9 | 6 |
|  | Status-off | 3 | 9 | 8 |
|  | | | | |
| *sabA* | Status-on | 3 | 7 | 6 |
|  | Status-off | 6 | 11 | 8 |
|  | | | | |
| *sabB* | Status-on | 4 | 10 | 4 |
|  | Status-off | 5 | 8 | 10 |

**Table 5.** Association between the grade of gastritis and the expression of outer membrane proteins in *Helicobacter pylori*.

| **Outer membrane protein** | | Grade of Gastritis | | |
| --- | --- | --- | --- | --- |
|  |  | Mild | Moderate | Marked |
| ***hopQ*** | allele 1 | 3 | 5 | 5 |
|  | allele 2 | 0 | 22 | 6 |
|  | | | | |
| *hopZ* | Status-on | 2 | 13 | 7 |
|  | Status-off | 1 | 14 | 4 |
|  | | | | |
| *oipA* | Status-on | 0 | 12 | 8 |
|  | Status-off | 3 | 15 | 3 |
|  | | | | |
| *sabA* | Status-on | 1 | 10 | 5 |
|  | Status-off | 2 | 17 | 6 |
|  | | | | |
| *sabB* | Status-on | 1 | 14 | 3 |
|  | Status-off | 2 | 13 | 8 |
